# Management of oligometastatic and oligoprogressive epidermal growth factor receptor mutated non-small cell lung cancer patients: state of the art of a combined approach

**DOI:** 10.37349/etat.2024.00228

**Published:** 2024-05-17

**Authors:** Francesca Di Pressa, Fabiana Perrone, Anna Benini, Frank Lohr, Marcello Tiseo, Alessio Bruni

**Affiliations:** Pathology Specialist at Hospital Clinic Barcelona, Spain; ^1^Radiation Therapy Unit, Department of Oncology and Hematology, University Hospital of Modena, 41124 Modena, Italy; ^2^Medical Oncology Unit, University Hospital of Parma, 43126 Parma, Italy; ^3^Proton Therapy Unit, APSS Trento and CISMed, University of Trento, 38100 Trento, Italy; ^4^Department of Medicine and Surgery, University Hospital of Parma, 43126 Parma, Italy

**Keywords:** Non-small cell lung cancer, radiotherapy, target therapy, epidermal growth factor receptor mutation, tyrosine kinase inhibitor, oligometastatic, oligoprogression

## Abstract

Recently, the development of targeted therapy approaches such as those based on tyrosine kinase inhibitor (TKI) greatly improved the clinical outcomes of patients affected by oncogene addicted advanced non-small cell lung cancer (NSCLC). Similarly, the improvement of radiation therapy techniques has permitted to deliver high radiation doses to a limited number of metastatic target lesions (oligopersistent or oligoprogressive), with limited high-dose normal tissue exposure that leads to low severe toxicity rates. The aim of this narrative review was to provide an overview of the currently established definition of oligometastatic and oligoprogressive disease, to define first line and subsequent lines targeted therapies and the role of consolidative non-invasive local ablative treatments (LATs) in these settings. The potential benefit of local treatment (LT) such as radiotherapy (RT) or surgery might be represented by an overall reduction of switching to subsequent systemic treatments lowering the risk of further systemic dissemination. Further randomized clinical trials will clarify the role of LT and their correct timing in relation to systemic targeted therapies.

## Introduction

Epidermal growth factor receptor (EGFR) mutations occur in almost 10–15% of patients affected by non-small cell lung cancer (NSCLC) in the western world and in 30–40% of the Asiatic population [1, 2]. These types of mutations are more common in non-smokers and females. At time of diagnosis, about 50% of patients present with advanced stage NSCLC, sometimes with an oligometastatic situation [3, 4]. The development of “targeted therapy” such as EGFR tyrosine kinase inhibitors (TKIs) dramatically improved the most important clinical outcomes when compared to historical doublet chemotherapy (CHT) [5]. EGFR TKIs are actually considered the standard first line therapy in patients with activating mutations of the EGFR exons 18–21. However, resistance to EGFR TKIs usually occurs after 9–13 months of first line with first-generations or second-generations agents. The most frequent tumor acquired alteration is represented by the T790M mutation that reduces the link of first-generation and second-generation TKIs to the ATP-binding pocket of EGFR with consequent progression of disease [6]. More recently, according to the results obtained in the phase III randomized FLAURA trial, osimertinib should be used as first-line systemic therapy regardless of T790M mutation status [7, 8] as it achieved a significant improvement in terms of both progression free survival (PFS) and even overall survival (OS).

*In vitro* and *in vivo* studies investigated the increasing sensitivity to radiotherapy (RT) of EGFR-TKIs. Ionizing radiation activates EGFR and mitogen-activated protein kinases (MAPK)-extracellular signal-regulated kinase (ERK) and phosphatidylinositol 3-kinase (PI3K)/protein kinase B (AKT) pathways causing the promotion of cellular proliferation and repair of radiation induced.

DNA damage through homologous and nonhomologous recombination [9]. The repeated exposition to radiation increased EGFR expression [9, 10]. EGFR-TKIs, such as gefitinib and erlotinib had a radiosensitizing effects at different levels: apoptosis, cell cycle arrest, repopulation and DNA damage [11]. EGFR-TKIs, combined with radiation, promote the reduction in S-phase fraction, the most radioresistant cell phase, increasing the cells number in G1 phase and G2 phase and enhance apoptosis induction [10].

Local treatment (LT), such as surgery, RT or thermal ablation, of primary or metastatic lesions in oligometastatic or oligoprogressive disease has been shown to obtain clinical benefits in EGFR mutated-NSCLC [12, 13]. The European Society for Medical Oncology (ESMO), suggest administering LT, such as high-dose RT or surgery, to no more than 3 synchronous metastatic sites in the oligometastatic setting after multidisciplinary discussion [13]. However, the management of oligoprogression is controversial due to the limited clinical data, and involves mainly radical LT, such as stereotactic body RT (SBRT) or surgery, to achieve disease control. In EGFR-mutant advanced NSCLC patients a common approach for treating oligoprogression is to continue the EGFR-TKI (that is controlling the greater proportion of the disease), while using local ablative therapy to eradicate the resistant clones in the area or areas of radiological progression. This therapeutic approach is supported by several, even if mainly retrospective, case series [12].

The concept of adding LT to systemic therapy comes from the idea that a metastasis directed LT approach may prolong the overall duration of the systemic treatment efficacy with a possible impact on clinical outcomes in select subgroups as already demonstrated in different solid tumors [14–16]. In the stereotactic ablative RT for the comprehensive treatment of oligometastases (SABR-COMET) basket trial patients with stage IV oligometastatic malignant neoplasms in whom controlled primary tumor and less than 5 metastases were diagnosed, SBRT to secondary lesions significantly improved median OS (28 months *vs.* 41 months). The treatment related toxicity was also satisfactory with grade 2 or superior side effects occurring in only 29% of patients; relevant grade 5 toxicity was reported only in 3% of the study population [17]. In a phase II trial, Iyengar et al. [18] evaluated the role of SBRT plus maintenance systemic therapy *vs.* maintenance CHT alone in patients with oligometastatic NCSLC (defined as six extracranial lesions, including primary site and less of 3 liver or lung lesions). The PFS was 9.7 months in the SBRT arm and 3.5 months after CHT alone without significative differences in terms of toxicities [18].

The aim of this literature overview is to analyze the role of the combination of metastasis directed therapy, focusing on RT, in combination with TKIs in patients with EGFR mutated oligometastatic and/or oligoprogressive advanced NSCLC.

## Oligometastatic/Oligoprogressive NSCLC: definition and features of different scenarios

For the first time in 1995, Hellman et al. [19] introduced the concept of a new “intermediate” clinical scenario between localized and advanced stage, defined as “the oligometastatic disease”: it is a clinical stage occurring during the natural clinical progression from a localized primary tumor to disseminated metastatic disease. For the authors, LTs focused on the few sites of oligometastatic localization were potentially able to improve clinical outcomes.

More recently, the European Society for Radiotherapy and Oncology (ESTRO) and OligoCare European Organisation for Research and Treatment of Cancer (EORTC) project identified several different scenarios [20]:

1) “*De novo*” oligometastatic disease: patients without other prior diagnosis of oligometastatic disease which is considered “synchronous” if the time interval between diagnosis of primary tumor and metastases is less than six months or “metachronous” if it is superior than six months.
2) “Repeat” oligometastatic disease: patients had a prior diagnosis of oligometastatic disease. To describe this clinical scenario, the authors propose a dynamic metastatic model in which it occurred after the failure of a primary systemic treatment administered for “*de novo*” oligometastatic disease.
3) “Induced” oligometastatic disease: patients had a history of polymetastatic disease and were submitted to first line systemic therapy. It is the disease stage, initially described by Hellman [19] when the CHT failed in controlling a few tumor cells that developed a strong resistance to systemic therapy.

The number of metastases needed to identify the “oligometastatic” stage is still debated. Lievens et al. [21] identified a number of 5 different metastatic sites as the upper boundary for defining a clinical stage as “oligometastatic”. For other authors, the definition of oligometastatic disease is not only a matter of number (of lesions), but also of limited regions [22] or organs [19] involved.

Identifying a clinical tumor stage as “oligometastatic” requires optimal imaging represented by 18-fluoro-deossi-glucose positron emission tomography (18-FDG PET) together with brain imaging (contrast enhancement magnetic resonance or computed tomography). This kind of imaging seems to detect those patients with more favorable prognosis, defined as patients with less than 5 site of metastases [3]. Regarding the localization of systemic lesions, brain, lung, adrenal gland, bone, liver and lymph nodes represent the most frequent sites of metastases [23].

Moving to the definition of the “oligoprogressive” scenario, the type and timing of detection of new or progressive lesions is the characterizing feature. It may be represented by single or few sites of progression, while the primary tumor is stable during treatment with systemic therapy (CHT, chemo-immunotherapy or immunotherapy alone) [24]. According to the ESMO clinical practice guidelines, oligoprogression in case of EGFR mutated disease can be defined as the progression of a small number of metastatic sites with stability of all the other systemic lesions during target therapy treatments [1]. In this scenario, it is supposed that a population of tumor cells acquired a sort of biological resistance to systemic therapy that can be classified as: receptor alteration, acquisition of bypass signaling pathway and phenotypic alterations [25].

Yang et al. [26] defined different patterns of progression: dramatic symptomatic progression with an increase of lesion size of more than 20% in less than 6 months; gradual progression if the volume of the metastatic lesions increases more than 20% in more than 6 months; local progression in case of a single extracranial progression. Depending on the type of progression, some observational studies seem to suggest the use of SBRT to eradicate resistant tumoral cell clones without discontinuation of TKIs with the aim of extending the same systemic therapy duration [1, 27].

## EGFR mutated advanced NSCLC: first-line therapies

EGFR-TKIs are the major first line therapy for patients with advanced NSCLC harboring activating EGFR mutation based on several phase III clinical trials exploring the efficacy of first, second and third generation EGFR-TKIs.

Gefinitib, erlotinib and icotinib are low molecular weight, reversible, oral first-generation EGFR inhibitors [28]. While gefinitib and erlotinib are Food and Drug Administration (FDA) and European Medicines Agency (EMA)-approved, icotinib is approved only in China. Both gefinitib and erlotinib have anilinoquinazoline-based structures and serve as ATP-competitors [29]. The second-generation EGFR-TKIs, including afatinib and dacomitinib, are irreversible pan-human EGFR/HER-1, HER-2 and HER-4 (ErbB) inhibitors which covalently bind to EGFR [30]. A broader inhibitory profile on the ErbB receptor family and a more robust inhibition of downstream signaling distinguish second generation TKIs from first generation EGFR-TKIs [31].

Several phase III trials have explored the efficacy of gefitinib (NEJ002, WJTOG-3405, IFUM, first-SIGNAL), erlotinib (EURTAC, OPTIMAL, ENSURE) and afatinib (LUX-Lung 3, LUX-Lung 6) [32]. A significant clinical benefit was observed for all the compounds in comparison with standard platinum-based CHT, both in terms of PFS, objective response rate (ORR) and disease control rate (DCR). In this regard, EGFR-TKIs granted a median PFS of 8.0–13.1 months and ORR of 62.1–75%, which was significantly better compared to standard platinum-based CHT [33].

Dacomitinib was compared to erlotinib and gefitinib in the “Advance Research for Cancer for targeted pan- HER therapy (ARCHER)” 1009 and ARCHER 1050 trials, respectively. Although dacomitinib showed more efficacy, the problematic safety profile limited the use in clinical practice [34, 35].

Outcomes of *EGFR* mutant metastatic NSCLC improved significantly with the introduction of third generation TKIs. Osimertinib, an oral irreversible EGFR-TKI, was compared to standard first-generation and second-generation EGFR-TKIs as a first-line treatment in the FLAURA trial. Remarkably, osimertinib was superior to both erlotinib and gefitinib in terms of PFS [18.9 months *vs.* 10.2 months, hazard ratio (HR, 0.46; *P* < 0.001)] [7], and OS [38.6 months *vs.* 31.8 months, HR 0.799; 95.05% confidence interval (CI), 0.641–0.997; *P* = 0.0462] [8]. Compared to early generation TKIs, osimertinib demonstrated a considerable survival benefit and a higher capacity to penetrate the blood brain barrier resulting in further central nervous system (CNS) response rates > 60%. Hence, as a result, osimertinib was approved in 2018 by both the FDA and EMA for the first-line treatment of NSCLC with activating EGFR mutations and it constitutes the standard treatment in this setting to date.

The use of first-generation and second-generation EGFR-TKIs is now limited in first line therapy, except afatinib which demonstrated a great activity against uncommon EGFR point mutations or duplications in exons 18–21, as showed in the post hoc analysis of LUX-Lung 02, LUX-Lung 03 and LUX-Lung 06 trials [36].

## EGFR mutated advanced NSCLC: beyond first-line

Despite EGFR-TKIs having led to a dramatic improvement in clinical outcomes in *EGFR* mutated NSCLC, the majority of patients eventually experience resistance to EGFR-TKIs, regardless of the lines of treatment.

The optimal second-line therapy for patients who progressed to first-line EGFR inhibitors depends mainly on the molecular mechanism of resistance. To offer the optimal therapy, a molecular recharacterization by tissue or liquid biopsy is strongly recommended in patients in which EGFR-TKIs failed as first-line treatment [37].

The most common resistance mechanism to first and second-generation EGFR-TKIs is the T790M mutation, occurring in up to 50–60% of cases. Osimertinib is the best treatment choice in this setting [38]. AURA 3 was a phase III clinical trial exploring osimertinib in patients having experienced progression disease after first-generation or second-generation EGFR-TKIs due to the onset of T790M resistance mutation. Compared to platinum plus pemetrexed CHT, osimertinib achieved a PFS of 10.4 months *vs.* 4.4 months (HR 0.30, *P* < 0.001) and an ORR of 71% *vs.* 31% [38]. In addition, osimertinib improved survival at 24 months and 36 months *vs.* CHT (55% *vs.* 43% and 37% *vs.* 30%, respectively), with a longer median OS (26.8 months *vs.* 22.5 months, respectively) [39]. Consequently, FDA and EMA approved osimertinib for the treatment of patients with metastatic EGFR-mutated NSCLC with an acquired T790M mutation after progression on previous EGFR-TKI therapy.

The mechanisms of resistance to osimertinib, regardless of the line of treatment, are various and are still under investigation. They can be classified in two different groups: *EGFR* dependent mechanisms and *EGFR* independent mechanism [37]. Among the first group, the C797S mutation is the most common occurring in 10–26% and 7% of patients treated with osimertinib as second-line and first-line treatment, respectively [38, 40]. In absence of a T790M mutation and in presence of a C797S mutation, patients may be treated with early EGFR inhibitors [41, 42]. When C797S appears in presence of a T790M mutation, the allelic context in which C797S is acquired with respect to T790M has potential implication for treatment. If C797S is found concomitantly (in trans) with T790M mutation, a combination of first-generation and third-generation EGFR-TKIs could be a valid choice. On the contrary, if the mutations are in cis, a resistance to all available EGFR-TKIs was reported and CHT is the best choice [43].

Moreover, other uncommon tertiary *EGFR* mutations (e.g., G796S, L792H, G724S, and L718X) that confer resistance to osimertinib, could be treated with early-generation EGFR-TKIs in the absence of T790M mutation [44].

The EGFR independent mechanisms of resistance to osimertinib include alternative signaling pathway activation or histological transformation.

Mesenchymal-epithelial transition (MET) amplification has been identified as the most common EGFR-independent mechanism of osimertinib resistance, accounting for 5–24% of cases [45]. A feasible choice to overcome the resistance driven by MET amplification is the combination of osimertinib with a MET inhibitor. Indeed, crizotinib in association with osimertinib showed clinical and radiological benefit [46, 47]. Other promising MET inhibitors in association with osimertinib are now under investigation [48]. Alterations in rat sarcoma (RAS)/MAPK and PIK3/AKT pathways, cell cycle gene alteration and oncogenic fusions have been identified as other mechanism of resistance [45].

After the failure of the treatment with osimertinib, novel agents such as human EGFR-3 (HER-3) or trophoblast cell surface antigen-2 (TROP-2) directed antibody-drug conjugates (ADC) exhibited promising results. Patritumab deruxtecan, an HER-3 ADC, or datopotamab deruxtecan, a TROP-2 targeting ADC, are being investigated for patients who were previously treated with osimertinib [48].

The transformation from adenocarcinoma to small cell lung cancer (SCLC) and squamous cell carcinoma (SCC) has been described after treatment with third-generation EGFR-TKIs like osimertinib as first-line or second-line [49–52]. Identification of histological transformation is imperative, due to the therapeutic and prognostic implications.

In absence of druggable mechanisms of resistance, CHT remains the standard of care. The addition of immune-check point inhibitors (ICIs) and anti-angiogenetic drugs was investigated in a subgroup of patients included in the IMpower150 trial, showing survival benefits in terms of both PFS and OS in the atezolizumab, bevacizumab, paclitaxel and carboplatin groups compared with the bevacizumab, paclitaxel and carboplatin ones [53, 54].

Finally, ICI monotherapy showed little clinical benefit in EGFR mutated NSCLC, thus its use in clinical practice is not recommended.

## TKIs combined with RT in EGFR mutated oligometastatic or oligoprogressive NSCLC patients: why and when

Oligometastatic disease is often approachable with local ablative treatments (LATs), such as SBRT or surgery. A retrospective study by Ashworth et al. [23] found a median 5-years OS of 29.4%, in patients treated on both the primary tumor and the metastatic lesions with radical intent, despite of type of treatment (surgery or SBRT).

Different characteristics should be considered in patients that are candidates for LATs: age, performance status, comorbidities, time of appearance of metastases (synchronous *vs.* metachronous), number of metastases, site of lesions (a better prognosis seems to be achievable in case of brain, lung or adrenal gland metastases), extension of primary tumor, mediastinal lymph node involvement (negative lymph nodes have a better prognosis) [55].

Park et al. [56] demonstrated that asymptomatic patients may obtain clinical benefits from the continuation of EGFR TKI treatment even if restaging had shown a radiological progression. In this clinical scenario, adding LATs to progressing metastatic sites should be considered, particularly if oligoprogression occurred [1]. For these reasons, aggressive LT with SBRT and/or surgery may help to extend the disease control interval [23].

Recently, Giraud et al. [57] tried to summarize all the possible choices of combined treatments and proposed a treatment scheme to be used in *EGFR* mutated oligometastatic/oligoprogressive patients treated with early generation TKIs:

1) Oligometastatic scenario (less than 5 lesions), synchronous:
  a. Extracranial metastases: LATs to all metastatic sites.
  b. Intracranial metastases: SBRT followed by TKI or TKI alone.
2) Oligoprogressive scenario (less than 5 lesions during TKI treatment):
  a. Oligoprogression: continue TKI, investigate for T790M mutation and administer LATs to all sites of radiological progression.
  b. Poli-progression: T790M investigation, if positive osimertinib or other second line therapies; if negative administer second line CHT doublet.
3) Oligopersistence (less than 5 lesions after at least 2 months of TKI): administer LATs to all sites of radiological persistence.

Similarly, Passaro et al. [58] recently completed the ESMO consensus statements on the management of EGFR mutant NSCLC patients confirming the clinical need of having data that are more consistent to be used in the daily clinic in the absence of randomized prospective trials. Even if in this consensus specific information on the use of LATs in this setting is not provided, several authors have suggested that SBRT may be used in case of oligoprogression during first or later TKI therapies with the aim of continuing the same treatments without increasing toxicities [58]. Similarly, in case of oligopersistence after a good response to systemic TKI therapy consolidating these results using metastasis directed SBRT with the aim of killing clonogen and TKI resistant cells (to primary tumor or systemic lesions) may be considered [58]. Different studies investigated the safety of the association between RT, on thoracic or metastatic sites, and EGFR TKIs. In a Chinese study, the combination of thoracic RT and TKIs for stage IV EGFR mutated NSCLC, found that grade 3 or superior adverse events were radiation pneumonitis and rash, frequently very well controlled by corticosteroids administration [59]. Jia et al. [60] found a high rate of grade 3 or superior pneumonitis (54%), including a Common Terminology Criteria for Adverse Events (CTCAE) grade 5, when palliative RT was associated to osimertinib. Zhou et al. [61] evaluated the safety of whole brain RT with or without EGFR TKIs. The combination of the treatments improves the ORR and 1-year survival rate. The treatment related rash was statistically higher in whole brain RT (WBRT) + TKI, but the incidence of nausea and vomiting, fatigue and myelosuppression were not different between two groups [61]. Guimond et al. [62] retrospectively analyzed the association between RT and EGFR TKI association. Erlotinib and gefitinib were reported safe with RT, but data with metastasis-directed or palliative RT are absent. The authors proposed to stop these systemic therapies 1–2 days before starting RT. They found a lack of data also for crizotinib and osimertinib, so authors recommended at least two days of suspension and also stressed the importance of lung dosimetry during the RT planning, especially for patients with interstitial lung disease [62].

### Role of LATs on oligometastatic patients

A retrospective analysis in oncogene driven NSCLC showed that the pattern of failure in patients treated with first line TKIs was equal to 49% at the primary site, 20.4% at distant site and 32.6% on both local and distant sites [63].

Due to these findings, a phase II trial analyzed the role of consolidative RT in patients affected by stage IV oligometastatic (3 or less lesions) NSCLC after first-line systemic therapy. Twelve percent of the enrolled population had EGFR mutations. Patients were randomized to receiving consolidative RT with or without maintenance treatment or maintenance treatment alone. The study was prematurely closed at the interim analysis because consolidative RT improved PFS in oligometastatic patients with stable disease after first line treatment (the probability in favor of the LAT arm was 99.46%) [22, 64]. At a median follow up of 38 months, PFS in the LAT group was 14.2 months *vs.* 4.4 months in the only-TKI maintenance group. OS was also superior in the LAT arm without severe toxicities higher than grade 3 [22]. Similarly, Hu et al. [65] conducted a retrospective study comparing patients who received EGFR-TKI alone and EGFR-TKI plus local consolidative therapy (surgery or RT). As in the prior study, median PFS was superior in patients treated with a combination of lesion directed therapy and systemic TKI (15 months *vs.* 10 months). As well as PFS, also OS was superior in the combination group compared to the maintenance EGFR TKI group (34 months *vs.* 21 months) [65].

Zeng et al. [66] analyzed the use of osimertinib in patients with residual oligometastases. Local consolidative treatments (LCT) such as surgery, RT or radiosurgery were performed during osimertinib treatment to the primary tumor or to metastatic lesions. One-year and 3-years PFS rates were 85.7%, 54.5% for patients submitted to LATs *vs.* 53.7% and 16.6% for patients treated with osimertinib alone [66].

Smith et al. [67] retrospectively analyzed severe acute lung toxicities in patients who received concomitant TKIs and RT. SBRT or intensity modulated RT were used with treatment planning based on four dimensional computed tomography. In terms of lung toxicities, 37.5% of patients developed grade 3 or superior pneumonitis. Analyzing more deeply potential predictive factors for developing pneumonia or RT-related pneumonitis, tumor location within 2 cm of the proximal bronchial tree seems to increase the risk of these kind of severe side effects [67].

The Asian phase III Stereotactic Body Radiation Therapy in Newly Diagnosed Advanced Staged lung adenocarcinoma (SINDAS) study enrolled 133 patients with oligometastatic *EGFR* mutated NSCLC, all receiving first generation TKIs therapy. The randomization was performed to obtain two different groups: one receiving RT to metastatic lesions and the other arm receiving only systemic treatment. Patients with brain metastases were excluded from the study. In the RT group, local RT treatments were performed to all metastatic lesions and to the primary tumor and involved intrathoracic nodal involved sites. Prescription dose between 25–40 Gy in 5 fractions were allowed. At time of last follow up, local control was 91.2% in the TKI + RT group and 55.4% in the TKI-only group. PFS and OS were respectively 20.2 months *vs.* 12.0 months and 25.5 months *vs.* 17.6 months, in the two arms. In the RT + TKI group 7.4% patients experienced grade 3–4 pneumonitis, 4.4% esophagitis; only one rib fracture was registered [68].

The NORTHSTAR phase II study enrolled patients with EGFR mutated stage IIIB–IV NSCLC and it has already completed accrual. Long terms results, however, are not yet available. Patients were randomized in two arms: the first one, receiving osimertinib for 8 weeks and local consolidation therapy (either surgery or RT) and the second receiving osimertinib alone. Primary endpoint was PFS at 4 years, secondary endpoints were OS, time to progression of target lesions, time to appearance of new metastases, PFS in oligometastatic group, toxicities [69].

Another issue that is still under investigation is the optimal timing between SBRT and systemic therapy with TKIs, for example for brain metastases (e.g., the ongoing OUTRUN study) [70]. This is an example of the several uncertainties regarding the management of brain metastases in EGFR mutated patients. It is known that TKIs are able to cross the blood-brain barrier and response rates of 80% in several trials have been observed, sometimes improving OS [7, 71]. For this reason, it is not clear if patients with treatable lesions using SBRT may benefit by an upfront approach (during the first 8 weeks of TKIs) or if it is sufficient to apply SBRT in those cases with further CNS oligoprogression or oligorecurrence.

Magnuson et al. [72] retrospectively analyzed patients with a new diagnosis of oligometastatic stage IV NSCLC with brain localizations never treated before. Patients were divided in 3 subgroups: 1—brain SBRT; 2—WBRT; 3—upfront TKI treatment. Patients receiving first line TKIs had less neurological symptoms and smaller lesions, while patients undergoing WBRT had the worst prognosis, a fact that is partially explained by selection bias as they had more than 10 lesions at time of diagnosis. Median OS for the three groups was 46 mouths, 30 mouths and 25 months respectively. At the multivariate analysis, SBRT was associated with improved OS in comparison to TKI only (HR 0.39, 95% CI 0.26–0.58, *P* < 0.001) [72]. Hence, the Spanish Society of Medical Oncology recommended the use of targeted therapy in patients with oligometastatic stage IV NSCLC with brain metastases, while LATs should be postponed, according to their guidelines [73]. Further data are needed to better clarify the overall picture, however, the principal abovementioned studies are summarized in Table 1.

**Table 1:** Summary of studies concerning oligometastatic EGFR mutated NSCLV

**Reference**	**Study**	**Period**	**Enrolled patients**	**Treatments**	***N* metastases; sites**	**Results (months)**	**Toxicity > 2**
Magnuson et al. [72], 2017	Retrospective	2008–2014	351	TKI +/– SRS or WBRT	1–10; brain	mOS 25, 46, 30	None
Gomez et al. [22], 2019	Randomized, phase II	2012–2016	49	TKI +/–consolidative RT	1–3; NS included brain	mPFS 14.2 *vs.* 4.4, OS 42.1 *vs.* 17.0	No grade > 3
Hu et al. [65], 2019	Retrospective	2010–2016	231	TKI +/– LCT	1–5; lung, bone, brain, others	mPFS 15.0 *vs.* 10.0, mOS 34.0 *vs.* 21.0	Not reported
Zeng et al. [66], 2020	Retrospective	2015–2019	108	Osimertinib +/– LCT	1–5; lung, bone, adrenal gland, brain, lymph nodes	mPFS 12.8 *vs.* NR mOS 85.8 *vs.* 77.1	Not reported
Khan et al. [69], 2022	Randomized		143	Osimertinib +/– LCT	1–3; NS	*	Not reported
Wang et al. [68], 2023	Randomized, phase III	2016–2019	133	1st generation TKI +/– RT	1–5; excluded brain	mPFS 20.2 *vs.* 12.5 mOS 25.5 *vs.* 17.4	6% G3–G4 pneumonitis (TKI + RT arm)

SRS: stereotactic radiosurgery; mOS: median OS; mPFS: median PFS; NS: not specified; +/–: with or without. ^*^ Trial on going

As LT, even surgery may have a role in the oligometastatic setting. Wang et al. [74] evaluated the role of surgery in patients with distant metastasis and compared lobectomy, pneumonectomy and sublobectomy finding that surgery may improve lung cancer specific survival (3-years 7.44% *vs.* 29.92%; 5-years 3.41% *vs.* 21.59%) and OS (3-years 6.58% *vs.* 27.57%; 5-years 2.89% *vs.* 18.87%). In a Japanese series [75], the authors reviewed the role of surgery on primary resection and bone metastases. They found that primary site resection and EGFR mutation patients had a longer OS (HR 4.18, 95% CI, 1.20–14.6, *P* = 0.025; HR 3.30, 95% CI, 1.08–10.1, *P* = 0.036).

### Role of LATs in oligoprogression during TKIs treatment

In the Canadian Consensus, the experts suggested that patients with limited oligoprogression during TKIs may receive LATs still staying on the same target therapy [13].

Different retrospective studies analyzed the role of metastasis directed RT in the oligoprogressive setting in *EGFR* mutated NSCLC patients. Xu et al. [76] retrospectively analyzed 206 patients treated with local ablative RT during first line TKI therapy. Brain metastases occurred in 60% of the patients, while bone metastases in 41.7%. Authors evaluated the “first PFS” defined as the first progression from starting TKIs to progression, the “PFS2” calculated from the start of TKI to its interruption and OS that were 10.7 months, 18.3 months and 37.7 months, respectively. One-year, 2-years and 3-years survival rate were 94.1%, 78.9% and 54.7%, respectively [76]. Furthermore, an Italian retrospective multicenter study analyzed 106 patients with oncogene driven mutations, 81% of whom had EGFR mutations and 49.1% had oligometastatic stage IV disease or experienced oligoprogression. SBRT or hypofractionated RT were administered concomitantly with TKIs. One-year and 2-years OS were 79% and 61.8%, respectively showing very interesting results in terms of clinical outcomes and safety [77].

In the study of Qiu et al. [78], 44 patients underwent a combination of RT and TKIs after having experienced oligoprogression. Two-year OS was 65.2% and mean OS was 35 months. Median OS and PFS were 13 months and 7 months, respectively [78].

In a retrospective matched cohort, SBRT was compared with CHT in patients with oligoprogressive *EGFR* mutated NSCLC. Fifty patients were enrolled, 25 received SBRT and 25 CHT alone. OS was better in patients who underwent SBRT (28.2 months *vs.* 14.7 months) as well as PFS (7 months *vs.* 4.1 months) [79].

Several studies also evaluated the importance of adding consolidative SBRT to osimertinib after progression on first-generation or second-generation TKIs in *EGFR* mutated patients. Authors suggested that adding an SBRT treatment to residual disease might have a significant role in improving time to progression (median PFS 17 months *vs.* 11 months) [80].

However, Jia et al. [60] highlighted that the combination of osimertinib and RT could be associated with an excess of severe toxicities, such as grade 2 pneumonitis in patients treated with thoracic RT and TKIs, even when a very low dose was delivered to critical organs at risks [81].

Finally, different scenarios can be considered in case of intracranial progression due to brain metastases. Shukuya et al. [81] analyzed 17 patients that received RT for brain metastases while continuing TKI systemic therapy. The median PFS, extracranical PFS and median OS were 80 days, 171 days, and 403 days, respectively [81]. Similarly, Weickhardt et al. [82] retrospectively analyzed patients with oncogene driven NSCLC with CNS oligoprogression during target systemic therapy. Twenty seven patients had *EGFR* mutations and received erlotinib and 85% had progression at time of analysis. LATs such as surgery or SBRT, RT or radiosurgery were performed after progression allowing almost six additional months of disease local control. No grade 3/4 side effects were registered. The most frequent RT related adverse event was fatigue [82]. Summarized results of these studies are shown in Table 2.

**Table 2:** Summary of studies concerning oligoprogressive EGFR mutated NSCLC

**Reference**	**Study**	**Period**	**Enrolled patients**	**Treatments**	**Results (months)**	**Toxicity > 2**
Shukuya et al. [81], 2011	Retrospective	2002–2009	17	TKI + RT to CNS lesions	mPFS 2.5, mOS 13.4	None
Weickhardt et al. [82], 2012	Retrospective	2005–2011	23	TKI + LAT	mPFS1 13.8, mPFS2 6.2	None
Qiu et al. [78], 2017	Retrospective	2009–2014	46	LT + TKI	mOS (after LT) 13.0, mPFS (after LT) 7.0, 2-years OS 65.2%	G3 pneumonitis 4.3%
Chan et al. [79], 2017	Retrospective	2013–2015	25	LAT *vs.* CHT	mOS 28.2 *vs.* 14.7, mPFS 7.0 *vs.* 4.1	1 G3 oesophagitis
Xu et al. [76], 2019	Retrospective	2011–2016	206	TKI + LAT	mPFS 10.7, mOS 37.4	G3 pneumonitis 1.5%
Borghetti et al. [77], 2019	Retrospective	2010–2016	106	RT concomitant/after TKI	mOS 23.0, 1-year OS 76.3%, 2-years OS 48.6%, 3-years OS 29.5%	3 G3 toxicity cases
Guo et al. [80], 2019	Retrospective	2017–2018	97	Osimertinib +/– SBRT (on residual disease after 1st/2nd PD)	mPFS 17.0 *vs.* 11.0	Not reported

mPFS1: median PFS from initiations to TKI to PD or death; mPFS2: median PFS from the time of first progression; PD: Progression Disease; SBRT: stereotactic body radiation therapy

According to ESMO consensus statements, third generation TKIs should be the first line therapy, also when leptomeninges are involved. In this last particular setting, the use of RT did not add significant benefits. Similarly, WBRT should be avoided, while SBRT can be considered in case of intracranial progression during TKI systemic therapy [58].

## Conclusions

In patients with EGFR mutated oligometastatic NSCLC, systemic therapy with third line EGFR inhibitor such as osimertinib represents the “gold standard” achieving very favorable results in terms of clinic outcomes when compared with CHT. However, many patients still develop loco-regional or systemic failure, often with a “slow” progression limited to few metastatic sites (oligoprogression or oligorecurrence). In this scenario, in well select patients, LT, such as SBRT or radiosurgery, should be considered. Indeed, LAT may be crucial to kill tumoral clones resistant to a given systemic therapy, to avoid systemic multiple sites dissemination and to stabilize the clinical state, permitting to continue the same systemic therapy. The limitations of this review are represented by its intrinsic not-systematic analysis of the literature, by the retrospective nature of most of the studies and by the non homogeneus population investigated. Furthermore, most of the presented studies, particularly those that are retrospective, do not clarify the timing of combination between RT and TKIs. Several studies had a satisfactory number of patients analyzed achieving very interesting results in terms of clinical outcomes. Finally, more prospective data are needed to better select candidates for consolidative LT without significant toxicity risk. This review may be useful to tailor combined therapy in patients clinical routine as it has been reported the current state of art in safely applying sophisticated personalized LT such as SBRT to metastatic oligoprogressive disease sites in the clinical practice. However, more robust data from clinical trials are needed to better clarify the role, the timing and the safety of metastasis directed local therapies and their impact in the daily routine.

## Abbreviations

ADC: antibody-drug conjugates
CHT: chemotherapy
CI: confidence interval
CNS: central nervous system
EGFR: epidermal growth factor receptor
EMA: European Medicines Agency
ESMO: European Society for Medical Oncology
FDA: Food and Drug Administration
HR: hazard ratio
LATs: local ablative treatments
LCT: local consolidative treatments
LT: local treatment
MET: mesenchymal-epithelial transition
NSCLC: non-small cell lung cancer
ORR: objective response rate
OS: overall survival
PFS: progression free survival
RT: radiotherapy
SBRT: stereotactic body radiotherapy
TKI: tyrosine kinase inhibitor

## Declarations

### Copyright

© The Author(s) 2024.
